# New data of spiders (Arachnida, Araneae) of Cyprus. 1. Dysderidae found in caves

**DOI:** 10.3897/zookeys.825.29029

**Published:** 2019-02-18

**Authors:** Salih Gücel, Iris Charalambidou, Bayram Göçmen, Kadir Boğaç K nt

**Affiliations:** 1 Institute of Environmental Sciences and Herbarium, Near East University, Nicosia, Cyprus Near East University Nicosia Cyprus; 2 Department of Life and Health Sciences, School of Sciences and Engineering, University of Nicosia, Cyprus University of Nicosia Nicosia Cyprus; 3 Department of Biology, Faculty of Science, Ege University, İzmir, Turkey Ege University İzmir Turkey; 4 Department of Biology, Faculty of Science, Eskişehir Technical University, Eskişehir, Turkey Eskişehir Technical University Eskişehir Turkey; 5 Zoological Collection of Cyprus Wildlife Research Institute, Taşkent, Kyrenia, Cyprus Zoological Collection of Cyprus Wildlife Research Institute Taşkent Cyprus

**Keywords:** Biospeleology, cavernicolous, island, Mediterranean, troglobiont

## Abstract

This paper is the first in a series describing the previously unstudied cave spiders from Cyprus. Two new species, *Dysderocrateskibrisensis***sp. n.** and *Harpacteakalavachiana***sp. n.**, are described. Detailed morphological descriptions and diagnostic characteristics are presented. This is the first report of the genus *Dysderocrates* Deeleman-Reinhold & Deeleman, 1988 from Cyprus.

## Introduction

The spider fauna of Cyprus, the third largest island of the Mediterranean, is poorly studied. One-hundred-fifteen species have been reported from the island (van Helsdingen 2018). The species diversity of the spider fauna on Cyprus, as compared to other Mediterranean islands, appears to be extremely low. For example, 555 species have been reported from Corsica, 523 from Sardinia, 421 from Sicily, 291 from Lesbos, 289 from Crete (van Helsdingen 2018), and approximately 315 species from Chios (Russell-Smith et al. 2011), an island in the Aegean that is a tenth the size of Cyprus. The distances of these islands to source landmasses, as well as human influence, are crucial for explaining this difference; however, araneological studies in Cyprus are extremely few and limited.

To fill this gap, we have begun a survey of the spider fauna of the island. Here we present our findings from previously uninvestigated caves. We found two new species of the dysderid genera *Dysderocrates* and *Harpactea*, and describe them based on females. Males were not sampled because they are only present for a short time in populations and because of the relatively low densities of cave spider populations in general.

## Materials and methods

**Figure 1.**
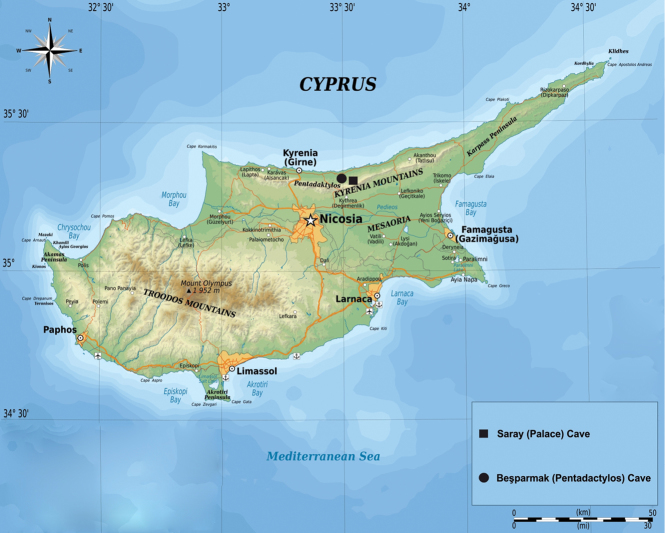
Collection localities.

Spider samples were collected from Beşparmak (Pentadactylos) and Saray (Palace) Caves (Fig. 1). Beşparmak Cave is approximately 500 m long and more than 200 m deep. The entrance is a small crack. After 5‒6 m of crawling, there is a 3 m drop, and after this point, the cave is completely dark. The spider was found 50 m from the entrance in the second chamber. The temperature in this cave was approximately 17 °C. Saray Cave is a smaller cave, approximately 40 m long. The entrance is wide, and the cave is dark after 15 m, and after large boulders at 20 m, the cave is completely dark. The spider was found approximately 25 m from the entrance.

Our specimens were preserved in 70% ethanol. Digital images of the copulatory organs were made with a Leica DFC295 digital camera attached to a Leica S8AP0 stereomicroscope. Between 5–15 photographs were taken at different focal planes and combined using Combine ZP (Hadley 2010). Terminology for the copulatory organs is adapted from Deeleman-Reinhold and Deeleman (1988), Deeleman-Reinhold (1993), and Chatzaki and Arnedo (2006). The number and disposition of spines follows the terminology of Özkütük et al. (2018). All measurements are given in mm.

The following abbreviations are used in the text and figures:

**Carapace and abdomen:**

AL abdominal length;

CL carapace length;

CWmax maximum carapace width;

CWmin minimum carapace width;

TL total length.

**Eyes:**

AME anterior median eyes;

PLE posterior lateral eyes;

PME posterior median eyes;

AMEd diameter of anterior median eyes;

PLEd diameter of posterior lateral eyes;

PMEd diameter of posterior median eyes.

**Chelicera:**

ChF length of cheliceral fang;

ChG length of cheliceral groove;

ChL total length of chelicera (lateral external view).

**Legs:**

Ta tarsus;

Mt metatarsus,

Ti tibia;

Pa patella;

Fe femur;

Tr trochanter;

C coxa;

d dorsal;

pl prolateral;

rl retrolateral;

v ventral.

**Depository:**

ETAM Eskişehir Technical University, Arachnology Museum (Eskişehir, Turkey);

NMNUNatural History Museum of Near East University (Nicosia, Cyprus);

ZMUTZoological Museum of the University of Turku (Turku, Finland).

## Taxonomy

### Family Dysderidae C.L. Koch, 1837

#### Genus Dysderocrates

Taxon classificationAnimaliaAraneaeDysderidae

Deeleman-Reinhold & Deeleman, 1988

##### Type species.

*Harpactocratesstorkani* Kratochvíl, 1935, from Macedonia.

#### Dysderocrates kibrisensis sp. n.

Taxon classificationAnimaliaAraneaeDysderidae

http://zoobank.org/676DA5F8-6327-4035-964F-491568DD3444

Figures 2–4 , 5–6

##### Type.

Holotype ♀ (NMNU); **CYPRUS**, Lefkoşa, Beşparmak Mountains, Beşparmak (Pentadactylos) Cave (35°17'22"N; 33°27'56"E), collected as a dead specimen inside the cave, ca 40 m from the cave entrance, 15.I.2018, leg. S. Gücel.

##### Comparative material.

Dysderocratescf.regina Deeleman-Reinhold, 1988: **TURKEY** 1♀ (ETAM), Konya, Beyşehir, Beyşehir Lake, Hacıakif Island (37°37'35.15"N; 31°28'55.66"E), 1183 m, 29.III.2011, leg. E.A. Yağmur (Fig. 7).

*Dysderocratestanatmisi* Karakaş Kılıç & Özkütük, 2017: **TURKEY** 1♀ (ETAM), Antalya, Elmalı, Göltarla Village (36°34'38"N; 29°55'49"E), *Cedruslibani* forest, under stones, 1065 m, 24.XII.2015, leg. K.B. Kunt & E.A. Yağmur (Fig. 8).

##### Derivatio nominis.

The specific name refers to the type locality “Kıbrıs”, which is the Turkish name of Cyprus.

##### Diagnosis.

*Dysderocrateskibrisensis* sp. n. can be differentiated from *D.silvestris* Deeleman-Reinhold, 1988 (spherical spermatheca) and *D.storkani* (pentagonal-shaped spermatheca) by the transverse spermatheca. The new species differs from *D.regina* by its longer spermatheca and triangular dorsal arch (*Da*, Fig. 7) and from *D.marani* (Kratochvíl, 1937) by its anchor-shaped spermatheca and the dorsal arch longer than spermatheca. The spermatheca of *D.kibrisensis* sp. n. is similar to those of *D.tanatmisi* but differs by the angular shape of the anterior part of the dorsal arch versus the semicircular shape of the same in *D.tanatmisi* (cf. Figs 5, 8).

##### Measurements of holotype.

TL 17.00; AL 9.00; CL 8.00; CWmax 6.00; CWmin 4.80; AMEd 0.29; PLEd 0.21; PMEd 0.20; ChF 2.00; ChG 1.20; ChL 3.70. Leg measurements as shown in Table 1.

**Table 1.** Leg measurements of *Dysderocrateskibrisensis* sp. n.

Leg	C+Tr	Fe	Pa	Ti	Mt	Ta	Total
**I**	4.50	9.50	5.50	8.20	8.00	1.25	36.95
**II**	4.00	8.40	5.00	7.00	7.50	1.20	33.10
**III**	3.00	6.50	3.50	5.00	6.50	1.20	25.70
**IV**	3.50	8.50	3.50	6.70	8.30	1.25	31.75

##### Description of holotype.

**Figures 2–4.**
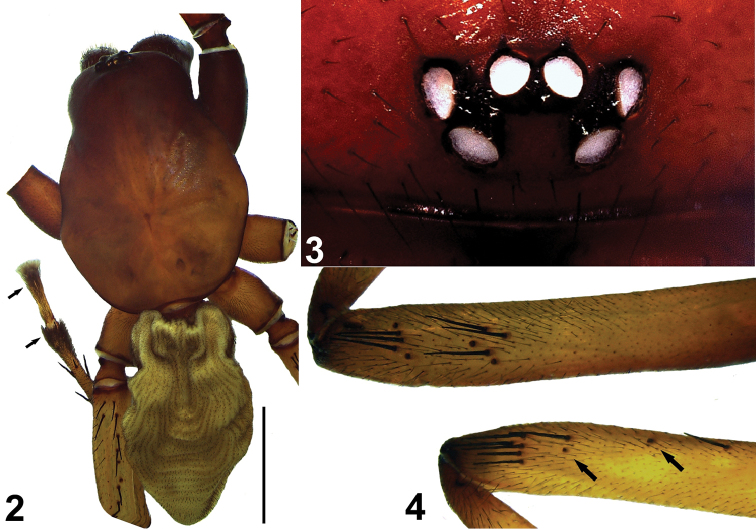
*Dysderocrateskibrisensis* sp. n. **2** Habitus of holotype female (arrows indicate tarsal and metatarsal scopulae) **3** Eyes **4** Femoral spination of anterior legs, left Leg I (above) right Leg II (below), prolateral view. Scale bar: 4 mm.

**Female.** Carapace red, smooth. Cephalic region much narrower and darker than thoracic region (Fig. 2).

Eyes well developed (Figs 2, 3). Chelicerae blackish red. Labium and gnathocoxae blackish. Sternum reddish. Anterior part of sternum darker than posterior. Legs reddish orange. Coxae and trochanters of legs I‒II darker than legs III‒IV. Spines clumped prolaterally on leg I, uniformly distributed along a line on leg II (Fig. 4). Leg spination as shown in Table 2.

**Table 2.** Leg spination of *Dysderocrateskibrisensis* sp. n.

♀	Fe	Ti	Mt
**I**	5, 5pl	0	0
**II**	1, 1, 2, 5pl	0	0
**III**	0–3d	2–3pl 2rl 1, 1 2v	4pl 3rl 1, 1, 2v
**IV**	8–10d	3pl 4rl 1, 1, 2v	5pl 7rl 1, 1, 2v

Tarsi and metatarsi III‒IV with scopulae. Scopulae in the first quarter of the metatarsi very dense (Fig. 2). Abdomen greyish-cream, covered with short, adpressed, dark setae arranged longitudinally (Fig. 2).

**Figures 5–8.**
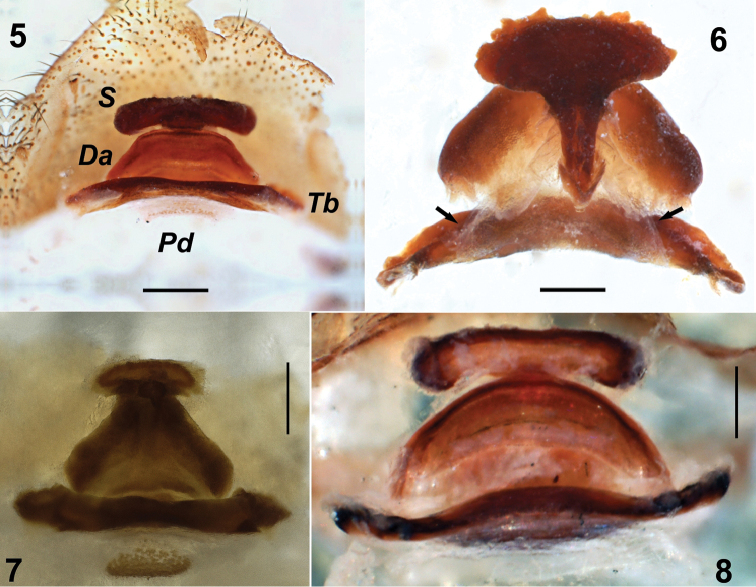
*Dysderocrateskibrisensis* sp. n. Vulva **5** dorsal view, arrow indicates triangular membranous structure **6** ventral view Dysderocratescf.regina Vulva **7** ventral view *Dysderocratestanatmisi* Vulva **8** ventral view. Abbreviations: ***Da*** Dorsal arch ***Pd*** Posterior diverticulum ***S*** Spermatheca ***Tb*** Transverse bar. Scale bars: 0.125 mm (**5–8**), 0.1 mm (**6**).

**Vulva.** Anterior spermatheca (*S*) and transverse bar (*Tb*) strongly sclerotized, dorsal arch (*Da*) relatively less sclerotized. Posterior diverticulum scarcely visible. Spermatheca flat with a button-shaped structure posteromedially. Anterior margin of spermatheca not smooth. Dorsal arch mushroom-cap-shaped. Dorsal arch (*Da*) and transverse bar (*Tb*) support triangular membranous structure. Transverse bar (*Tb*) arched (Figs 5, 6).

Male unknown.

##### Distribution.

Known from the type locality only.

##### Comments.

Seven species of *Dysderocrates* are known, and the entire genus is restricted to the Mediterranean Basin. We placed this species in *Dysderocrates* because it fits the diagnosis: large body size, three strong teeth on the cheliceral groove, and many spines on the anterior femora. *Dysderocrateskibrisensis* sp. n. is the first and only species of the genus reported from Cyprus.

#### Genus Harpactea

Taxon classificationAnimaliaAraneaeDysderidae

Bristowe, 1939

##### Type species.

*Araneahombergi* Scopoli, 1763.

#### Harpactea kalavachiana sp. n.

Taxon classificationAnimaliaAraneaeDysderidae

http://zoobank.org/45D4FDDE-3953-425D-97F8-F701CACF0957

**Figures 9–12.**
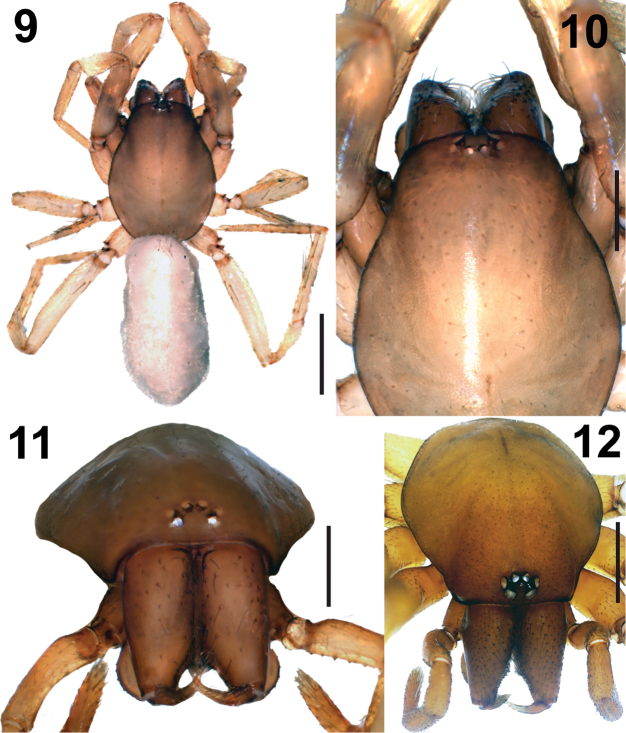
*Harpacteakalavachiana* sp. n. **9** Habitus of holotype female **10** Carapace, dorsal view **11** Carapace, anterior view **12** Anterior view of *H.alanyana*, female.

**Figures 13–16.**
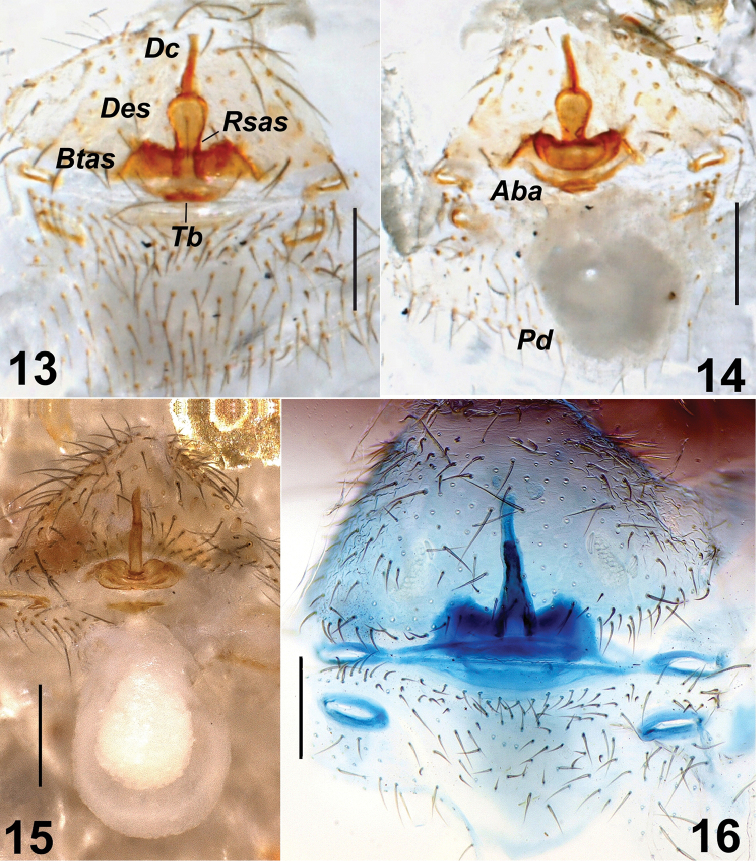
*Harpacteakalavachiana* sp. n. Vulva **13** ventral view **14** dorsal view *H.alanyana* Vulva **15** dorsal view *H.parthica* Vulva **16** ventral view. Abbreviations: ***Aba*** Anterior basal arc ***Btas*** Basal transverse part of the anterior spermatheca ***Dc*** Distal crest ***Des*** Distal expansion of the spermatheca ***Pd*** Posterior diverticulum ***Rsas*** Rod shaped part of the anterior spermatecha ***Tb*** Transverse bar. Scale bar: 0.2 mm.

Figures 9–11 , 13 , 14

##### Type.

Holotype ♀ (NMNU); **CYPRUS** Lefkoşa, Kalavaç Village, Saray (Palace) Cave (35°16'56"N; 33°32'09"E), hand collecting, 521 m, 14.VII.2017, leg. K.B. Kunt.

##### Comparative material.

*Harpacteaalanyana* Özkütük, Elverici, Marusik & Kunt, 2015: **TURKEY** 1♀ (ETAM) Antalya, Alanya, Taşatan Plateau (36°38'37.35"N; 32°4'42.09"E), 24.IV.2011, leg. R.S. Özkütük (Figs 12, 15).

*Harpacteaparthica* Brignoli, 1980: **IRAN** 1♀ (ZMUT), Mazandaran, 16.IX.1971, leg. P.T. Lehtinen & K. Kavén (Fig. 16).

##### Derivatio nominis.

The specific name refers to the type locality.

##### Diagnosis.

The general appearance of the broad posterior diverticulum, short transverse bar, and basal transverse part of the the anterior spermathecae of *H.kalavachiana* sp. n. are similar to those of *H.alanyana* (Turkey) (Fig. 15), *H.parthica* (Iran) (Fig. 16), and *H.digiovannii* Gasparo, 2014 (Cyclades, Greece). However, the distal expansion of the spermathecae in *H.kalavachiana* is 4‒5 times broader than the aforementioned species. *Harpacteakalavachiana* sp. n. differs from *H.gunselorum* Gücel, Fuller, Göçmen & Kunt, 2018 from Cyprus by the enlarged distal expansion of the spermatheca which is more than twice as wide as that of *H.gunselorum*, and from *H.cecconi* (Kulczyński, 1908) by its body length, which is larger (female of *H.cecconi*: 5.15 mm).

##### Measurements of holotype.

TL 3.00; AL 1.70; CL 1.30; CWmax 1.00; CWmin 0.48; AMEd 0.03; PLEd 0.02; PMEd 0.02; ChF 0.31; ChG 0.15; ChL 0.78. Leg measurements as shown in Table 3.

**Table 3.** Leg measurements of *Harpacteakalavachiana* sp. n.

Leg	C+Tr	Fe	Pa	Ti	Mt	Ta	Total
**I**	0.60	1.00	0.64	1.40	0.56	0.24	4.20
**II**	0.60	0.92	0.50	0.75	0.66	0.24	3.67
**III**	0.38	0.80	0.33	0.50	0.50	0.30	2.81
**IV**	0.50	1.10	0.45	0.88	1.00	0.30	4.23

##### Description of holotype.

**Female.** Carapace light brown. Cephalic region narrower and lighter compared to the thoracic region. Carapace with short blackish setae, fovea distinct. Eyes reduced (Figs 10–12).

Chelicerae and cheliceral fangs light brown. Chelicerae with scattered setae of varying lengths on the anterior surface (Figs 9–11). Labium, gnathocoxae, and sternum milky brown. Sternum with more hairs on the edges compared to the middle. Legs greyish. Coxae, trochanters, and especially the femora of legs I and II are darker than others (Fig. 9). Leg spination shown in Table 4.

**Table 4.** Leg spination of *Harpacteakalavachiana* sp. n.

♀	C	Fe	Pa	Ti	Mt
**I**	0	2pl	0	0	0
**II**	0	1, 1pl	0	0	0
**III**	1d	1, 1pl 1, 1d 1, 1rl	1rl	2, 2pl 2, 2rl 1, 1, 2v	1pl 1, 1rl 1, 1, 2v
**IV**	1d	2–3d	0	4pl 1, 1, 1rl 5v	1, 1, 1pl 1, 1, 1rl 4v

Legs III and IV with weakly developed scopulae on distal parts of tarsi and metatarsi. Abdomen cylindirical, grey-brownish (Fig. 9). Abdomen covered with short, greyish setae, anterior setae longer than the ventral and dorsal ones. Margins of the tracheal spiracles are slightly sclerotized.

**Vulva.** Anterior part of vulva sclerotized. Distal crest (*Dc*) spinose. Distal crest (*Dc*) and the length of the rod-shaped part of the anterior spermatheca (*Rsas*) subequal in length. The width of the distal expansion of the spermatheca (*Des*) about twice longer than rod-shaped part of the anterior spermatheca (*Rsas*). Transverse bar (*Tb*) short and straight. Posterior diverticulum (*Pd*) well developed (Figs 13, 14).

Male unknown.

##### Distribution.

Known from the type locality only.

##### Comments.

*Harpactea* is the second largest genus of the Dysderidae with 181 named species. Most *Harpactea* species have six well-developed eyes, although several cave-dwelling species exhibit different levels of eye reduction. *Harpacteasanctidomini* Gasparo, 1997 (Tremiti Islands, Italy) has only four eyes, with the PME entirely reduced. *Harpacteapersephone* Gasparo, 2011 (Kournas Cave, Chania Prefecture, Crete, Greece), *H.karaschkhan* Kunt et al., 2016 (Yalandünya Cave, Gazipaşa, Antalya, Turkey), *H.stalitoides* Ribera, 1993 (Iberian Peninsula), and *H.strinatii* Brignoli, 1979 (Diros Caves, Peloponnese, Greece) are eyeless.

The eyes of *H.kalavachiana* sp. n. are reduced, and the AME are distant from each other, much more than average (Fig. 11). Epigean species of *Harpactea* are usually reddish, however, *H.kalavachiana* is paler compared to the other epigean species of *Harpactea* (Fig. 12).

*Harpacteakalavachiana* sp. n. can be considered part of the *rubicunda* (D) species group according to the grouping by Deeleman-Reinhold (1993) due to the large, membranous posterior diverticulum and the spination of the coxae and patellae.

## Results and discussion

The results of our study increase the number of dysderid species on Cyprus from seven to nine, which increases the entire spider fauna to 117. Because the distributions of the two new species are limited to their type localities and because most cave species have small distributions, it is very likely that they are endemic to Cyprus.

Our arachnological sampling on the island continues. Future additional samples, including males of these two new species, will help determine their generic position more precisely.

## Supplementary Material

XML Treatment for Dysderocrates

XML Treatment for Dysderocrates kibrisensis

XML Treatment for Harpactea

XML Treatment for Harpactea kalavachiana
